# High-throughput single-microbe RNA sequencing reveals adaptive state heterogeneity and host-phage activity associations in human gut microbiome

**DOI:** 10.1093/procel/pwae027

**Published:** 2024-05-23

**Authors:** Yifei Shen, Qinghong Qian, Liguo Ding, Wenxin Qu, Tianyu Zhang, Mengdi Song, Yingjuan Huang, Mengting Wang, Ziye Xu, Jiaye Chen, Ling Dong, Hongyu Chen, Enhui Shen, Shufa Zheng, Yu Chen, Jiong Liu, Longjiang Fan, Yongcheng Wang

**Affiliations:** Department of Laboratory Medicine of The First Affiliated Hospital & Liangzhu Laboratory, Zhejiang University School of Medicine, Hangzhou 310058, China; Key Laboratory of Clinical In Vitro Diagnostic Techniques of Zhejiang Province, Hangzhou 310058, China; Institute of Bioinformatics, Zhejiang University, Hangzhou 310058, China; Department of Laboratory Medicine of The First Affiliated Hospital & Liangzhu Laboratory, Zhejiang University School of Medicine, Hangzhou 310058, China; Department of Laboratory Medicine of The First Affiliated Hospital & Liangzhu Laboratory, Zhejiang University School of Medicine, Hangzhou 310058, China; Key Laboratory of Clinical In Vitro Diagnostic Techniques of Zhejiang Province, Hangzhou 310058, China; M20 Genomics, Hangzhou 310058, China; M20 Genomics, Hangzhou 310058, China; M20 Genomics, Hangzhou 310058, China; M20 Genomics, Hangzhou 310058, China; Department of Laboratory Medicine of The First Affiliated Hospital & Liangzhu Laboratory, Zhejiang University School of Medicine, Hangzhou 310058, China; Department of Laboratory Medicine of The First Affiliated Hospital & Liangzhu Laboratory, Zhejiang University School of Medicine, Hangzhou 310058, China; M20 Genomics, Hangzhou 310058, China; Institute of Bioinformatics, Zhejiang University, Hangzhou 310058, China; Institute of Bioinformatics, Zhejiang University, Hangzhou 310058, China; Department of Laboratory Medicine of The First Affiliated Hospital & Liangzhu Laboratory, Zhejiang University School of Medicine, Hangzhou 310058, China; Key Laboratory of Clinical In Vitro Diagnostic Techniques of Zhejiang Province, Hangzhou 310058, China; Department of Laboratory Medicine of The First Affiliated Hospital & Liangzhu Laboratory, Zhejiang University School of Medicine, Hangzhou 310058, China; Key Laboratory of Clinical In Vitro Diagnostic Techniques of Zhejiang Province, Hangzhou 310058, China; M20 Genomics, Hangzhou 310058, China; Institute of Bioinformatics, Zhejiang University, Hangzhou 310058, China; Department of Laboratory Medicine of The First Affiliated Hospital & Liangzhu Laboratory, Zhejiang University School of Medicine, Hangzhou 310058, China

**Keywords:** single-microbe RNA sequencing (smRNA-seq), droplet microfluidics, microbiome, host-phage association, smRandom-seq2

## Abstract

Microbial communities such as those residing in the human gut are highly diverse and complex, and many with important implications for health and diseases. The effects and functions of these microbial communities are determined not only by their species compositions and diversities but also by the dynamic intra- and inter-cellular states at the transcriptional level. Powerful and scalable technologies capable of acquiring single-microbe-resolution RNA sequencing information in order to achieve a comprehensive understanding of complex microbial communities together with their hosts are therefore utterly needed. Here we report the development and utilization of a droplet-based smRNA-seq (single-microbe RNA sequencing) method capable of identifying large species varieties in human samples, which we name smRandom-seq2. Together with a triple-module computational pipeline designed for the bacteria and bacteriophage sequencing data by smRandom-seq2 in four human gut samples, we established a single-cell level bacterial transcriptional landscape of human gut microbiome, which included 29,742 single microbes and 329 unique species. Distinct adaptive response states among species in *Prevotella* and *Roseburia* genera and intrinsic adaptive strategy heterogeneity in *Phascolarctobacterium succinatutens* were uncovered. Additionally, we identified hundreds of novel host-phage transcriptional activity associations in the human gut microbiome. Our results indicated that smRandom-seq2 is a high-throughput and high-resolution smRNA-seq technique that is highly adaptable to complex microbial communities in real-world situations and promises new perspectives in the understanding of human microbiomes.

## Introduction

The human microbiome is made up of a huge number and a large variety of microbes in the gastrointestinal tract and has significant links to various human health- and disease-related conditions (Natarajan et al., 2020; Sharma et al., 2018). The behavior and biological effects of a microbial community are determined not only by its species composition and diversity but also by the cell states that occur within each microbe. The bacterial cell states are heavily influenced by the gene transcriptional activity of each microbe included in the community (Chong et al., 2014). For instance, previous studies reported that *Escherichia coli* has distinct persistence phenotypes after antibiotic treatment, which was caused by the heterogeneity of gene transcriptional activity (Roemhild et al., 2022). Variations in bacterial transcriptional states within subpopulations can result in differences in essential traits such as antibiotic resistance and metabolic capabilities (Smith et al., 2023), which can have a significant impact on human health. Therefore, it is necessary to adopt further approaches to achieve a complete functional characterization of host-associated microbes, given the well-known functional heterogeneity among populations of bacteria.

The advancement of single-cell RNA sequencing and its successful application in mammalian systems in the last decade has shed light on the tremendous transcriptional heterogeneity of cell types and states and transformed biological research in multiple fronts (Han et al., 2021; Klein et al., 2015; Paik et al., 2020; Papalexi et al., 2018; Van de Sande et al., 2023; van der Leun et al., 2020). Of note, several bacterial scRNA-seq methods [for instance microSPLiT (Kuchina et al., 2021), PETRI-seq (Blattman et al., 2020), MATQ-seq (Imdahl et al., 2020), par-seqFISH (Dar et al., 2021), ProBac-seq (McNulty et al., 2023), BacDrop (Ma et al., 2023), smRandom-seq (Xu et al., 2023a)] have been developed recently. Nevertheless, among several major limitations, the current microbial techniques are limited to well-characterized taxa and still not applicable to clinical- and physiology-relevant human microbiome samples yet. Consequently, the studies using the existing methods only looked at population heterogeneity in few well-characterized bacteria, such as artificial lab-generated culture mix. Therefore, a single-microbe RNA sequencing (smRNA-seq) technique for poorly-characterized and complex microbial communities in the world is critically needed.

In this study, we introduce smRandom-seq2, a high-throughput and high-resolution smRNA-seq method that is highly adaptable to complex microbial communities. Through the analysis of four fecal samples collected from healthy human subjects, we successfully obtained 29,742 single microbe barcodes from the gut samples. We further developed an analysis pipeline for microbe annotation and bacteria-phage transcriptional activity in the complex microbial community. All in all, we demonstrate that smRandom-seq2 is a novel sequencing technique for investigating single microbe transcriptional activity and can provide insights into the functional heterogeneity and interplay between bacteria and bacteriophages in the human gut microbiome.

## Results

### Overview of the smRandom-seq2 technique

**Figure 1.**
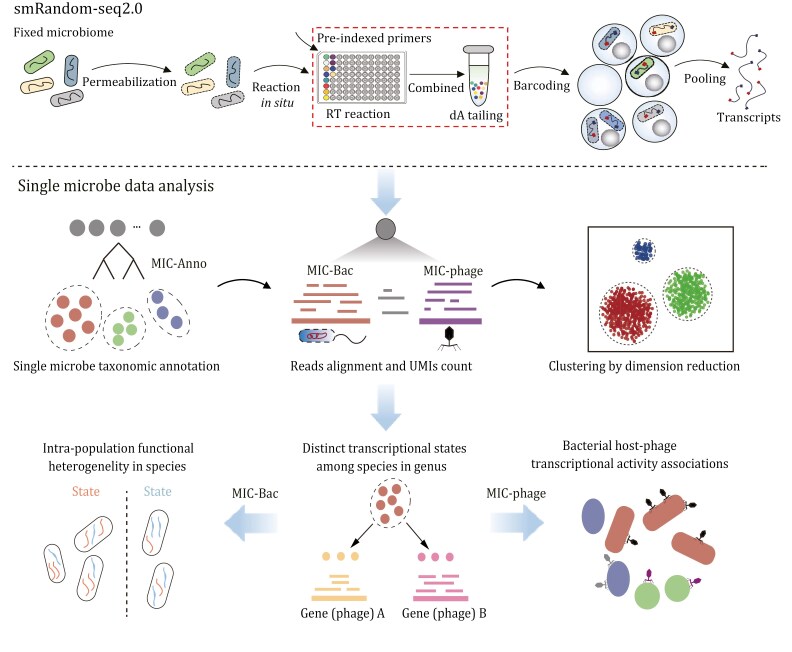
Overview of smRandom-seq2 and data analysis. First part is experimental methodology and workflow of smRandom-seq2. The second part is a single microbe data analysis pipeline for smRandom-seq2 data. After taxonomic annotation (MIC-Anno), each cell (barcode) will be assigned a taxonic information, and further clustered and heterogeneity and host-phage association analysis using MIC-Bac and MIC-Phage.

The gut microbiome presents unique difficulties for smRNA-seq due to its complexity and low efficiency of RNA capture for diverse species. To increase the efficiency of reverse transcription for all species and reduce cross-contamination, we designed a set of random primers with “GAT” three nucleotides and pre-indexes based on previous works (Sheng et al., 2017; Xu et al., 2023b). The fixed bacteria were evenly divided into 12 tubes for reverse transcription (RT) reaction with the pre-indexed random primers (Fig. 1). The underlying basis for the primer optimization in smRandom-seq2 is that the composition of human microbial community is much more complicated than cultured bacteria, which causes the capture efficiency of common random primers significantly decreased, leads to the bacteria capture bias and results in a low number of species and genes detected in the microbial community samples. So we systematically optimized the random primer in smRandom-seq2. We have screened different random and semi-random primer designs, and a set of GAT random primers with the highest efficiency was ultimately selected. And the results showed that the novel design of GAT random primers named PIX-1 showed the highest cDNA yield (Fig. S1A, the 12 random primers are shown in Tables S1 and S2).

Subsequently, a poly (dA) tailing step was performed to add poly(A) tails on the 3ʹ side of the cDNAs inside the bacteria. To effectively barcode individual bacteria, we developed an automated droplet collection system to generate smaller droplets with smaller poly (T) barcoded beads than the preceding inDrop platform wherein droplets were ~240 μm and beads measured around ~60 μm, our study adopted diminutive dimensions of droplets (~80 μm) and beads (~40 μm) (Fig. S2, the barcoded primers are shown in Table S2). By utilizing smaller droplets, we effectively increase the bacteria count within each droplet of the same volume. In addition, the reduced volume of smRandom-seq2 leads to higher cDNA concentrations in the reaction systems. This system allowed for the efficient generation of unique barcodes for each bacterium’s cDNAs and will help to increase the capture efficiency in smRandom-seq2. After barcoding, the cDNAs were pooled and PCR amplified for sequencing.

Analysis methods for scRNA-seq data in mammalian systems have been well-developed in recent years. However, these methods could not be applied to smRNAs from a complex microbial community for several reasons: (1) Current scRNA analysis methods were all based on a few species with well-annotated reference genomes. However, in the microbiome samples, various bacteria species were included in one smRNA dataset. It is difficult for the gene expression level quantification in each single microbe by a high-quality genome and its gene annotation. (2) Diverse bacteria exist in a microbial community. Even with the gene expression generated in each single microbe, how to integrate the expression data from multiple bacteria species is still a challenge. (3) Relationships between bacteria and phages in the human gut were mainly statistically speculated using the metatranscriptomic datasets up to now and no method could directly dissect the host-phage associations in smRNA-seq data. To solve these problems, we developed a computational pipeline, which contained three modules, single microbe annotation (MIC-Anno), the bacteria (MIC-Bac), and phage (MIC-Phage) transcriptional activity analysis, for smRNA-seq dataset from complex microbial community (Fig. 1). By the above pipeline, we are able to obtain accurate taxonomic information for each microbe and further bacterial and host-phage association analysis from the smRNA-seq (smRandom-seq2) data.

### Validation of smRandom-seq2 performance using mock microbial communities

**Figure 2.**
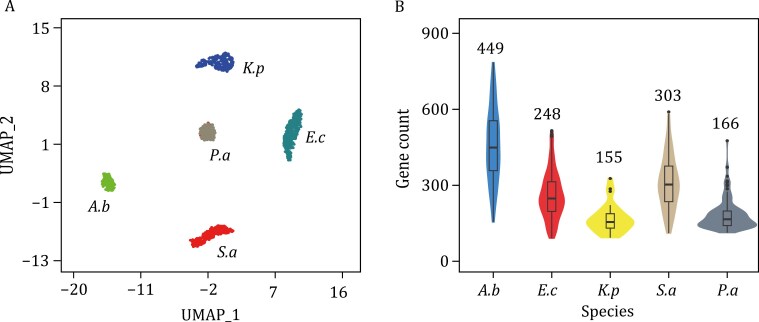
Performance of smRandom-seq2 on a mock microbial community with five known species. (A) UMAP plot of the smRandom-seq2 cells and clusters colored by species. (B) Distribution of gene count of the five bacterial species clusters by smRandom-seq2. The median gene count of each species is presented at the top of the violin plot. The mock community includes *A*. *baumannii* (*A*. *b*), *E*. *coli* (*E*. *c*), *K*. *pneumonia* (*K*. *p*), *P*. *aeruginosa* (*P*. *a*), and *S*. *aureus* (*S*. *a*).

We conducted the smRandom-seq2 assay on a mock community consisting of both gram-negative bacteria (*E*. *coli*, *Klebsiella pneumoniae*, *Acinetobacter baumannii*, and *P*. *aeruginosa*) and gram-positive bacteria (*Staphylococcus aureus*) to validate the optimized methods. Prior to microfluidic encapsulation, we confirmed the single bacterial morphology and manually counted the bacteria under a microscope. We evaluated the efficiency of droplet barcoding under a microscope, and assessed the quality of the resulting cDNA library using electropherograms. During the loading procedure, the number of loaded cells into the mocrofluidic device was ~10,000, about 50 μL droplets containing 2,500 cells were used for further experiments. Finally, we recovered 1,103 cells (~44% cell recovery rates) with the expression of a minimum of 98 genes per cell. The obtained bacterial library was further sequenced and their RNA profilings were visualized with Uniform Manifold Approximation and Projection (UMAP) dimensionality reduction and displayed clearer separation (Fig. 2A). The results demonstrate that smRandom-seq2 efficiently captured mRNA from each bacterial species, with an average median count of 260 genes per cell for *A*. *baumannii* (449), *E*. *coli* (248), *S*. *aureus* (303), *K*. *pneumoniae* (155), and *P*. *aeruginosa* (166) (Fig. 2B). The median purity of each species is between 0.90 and 0.99 (Fig. S3D). The technical repeatability of the smRandom-seq2 was verified by high correlation (*R* = 0.90, *P* < 2.2 × 10^−16^) on gene expressions among replicates (Fig. S3G). In addition, our analysis results showed that only 0.9% of the sequenced reads were mapped to the non-coding regions in the genome, which is significantly lower than the total non-coding regions percentages (11.26%) in the reference genome. These results demonstrated that little DNA contamination existed in smRandom-seq2.

### Transcriptional activity landscape of individual bacterium in a human gut microbiome

**Figure 3.**
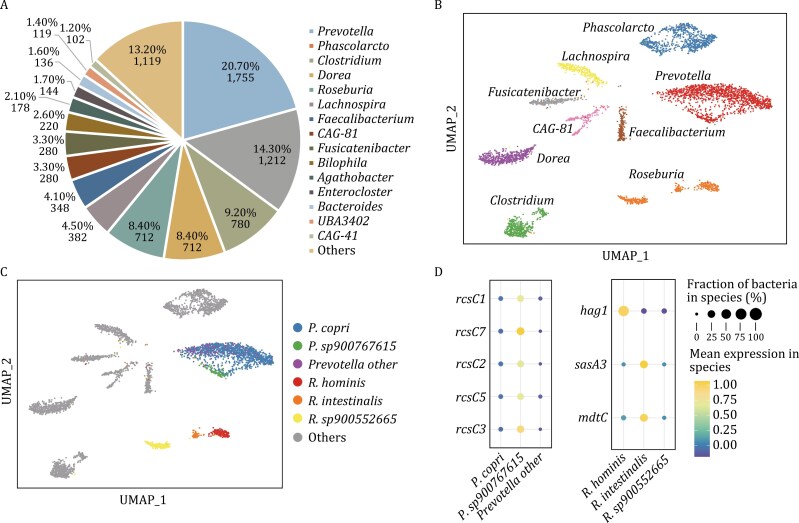
Bacterial gene expression landscape in a human gut microbiome. (A) Bacteria proportion of each genus identified in the smRandom-seq2 dataset. (B) UMAP plot of the smRandom-seq2 cells and clusters colored by genus taxonomic annotation using MIC-anno. (C) UMAP plot of the clusters colored by species of *Prevotella* and *Roseburia* genus. (D) Dot plot of significantly up-regulated genes in species in the *Prevotella* (left) and *Roseburia* (right) clusters.

We first proceeded to apply the smRandom-seq2 technology to a human fecal sample from a healthy donor. In total, 8,478 cells were captured from the sample and an average depth of 12,782 reads per cell (unique barcode). As the human gut microbiome contained many bacterial species, we first applied MIC-Anno for bacterial annotation in the gut dataset. Based on the annotation results, we identified 98 species in the gut microbiome. Among them, 15 genera were a higher abundance than 1% (Fig. 3A) and the top five abundant genera were *Prevotella*, *Phascolarctobacterium*, *Clostridium*, *Dorea*, and *Roseburia*. To validate the bacterial percentages or composition, we also performed metatranscriptome sequencing on the same gut samples and calculated the species abundance using MetaPhlAn2. The results showed that the bacterial abundance by smRandom-seq2 was significantly correlated with metatranscriptome sequencing (*R* = 0.98, *P* < 0.001), suggesting that smRandom-seq2 could capture different bacterial species in the gut microbiome without obvious bias (Fig. S4A). To investigate whether there was mixed contamination between barcodes in the smRandom-seq2, we further evaluate the purity (reads percentage of the corresponding annotation taxon) of each barcode in different genera (Fig. S4B) and the results indicated that most of the barcodes had a high purity (> 90%). At the species level, the median purity of each species is between 0.89 and 0.95.

We further used MIC-Bac to acquire single bacterial gene expression data of the gut microbiome for the smRandom-seq2 data. The proportion of rRNA in this microbiome sample is 75.30%. Upon analysis subsequent to rRNA removal, each bacterium, on average, encompasses 3,158 non-rRNA reads. Despite the absence of rRNA depletion during sequencing, the quantity of non-rRNA sequences within the samples is sufficient to facilitate subsequent analysis of bacterial gene functions. After removing rRNA genes, for each genus in the gut microbiome, we detected 170, 186, 386, 167, 209, 211, 128, 272, and 218 median genes per cell in genera *Prevotella*, *Phascolarctobacterium*, *Clostridium*, *Dorea*, *Roseburia*, *Lachnospira*, *Faecalibacterium*, CAG-81, and *Fusicatenibacter*, respectively (Fig. S4C). The most highly detected expressed genes encode ribosomal proteins and translation elongation machinery. Based on the integrated gene expression count matrix of all barcodes, 11 cell clusters or the gut microbiome transcriptomic states were grouped (Fig. S4D). Based on the taxonomic annotation results, the 11 cell clusters were assigned to 9 bacterial genera which included *Prevotella*, *Clostridium*, *Fusicatenibacter*, *Dorea*, CAG-81, *Roseburia*, *Phascolarctobacterium*, *Faecalibacterium*, and *Lachnospira* (Fig. 3B). At last two clusters were included in the *Prevotella* genus (Cluster #0, #10) and *Roseburia* genus (Cluster #6, #9), respectively. To investigate the potential functional heterogeneity in these two genera, we further annotated the *Prevotella* and *Roseburia* clusters at species level.

### Distinct adaptive response states among species in the human gut microbiome

In the *Prevotella* genus, a total of 1,755 cells were identified as 11 *Prevotella* species, most being *P. copri* (79%) and *P. sp900767615* (7.2%), which corresponded to Cluster #0 and Cluster #10, respectively (Fig. 3C). *P*. *copri* is a well-known dominant bacterial species in the human gut (Prasoodanan et al., 2021). To further validate the annotation results, we assembled the transcriptome based on the reads in all barcodes of corresponding species. The assembled transcripts of *P*. *copri* and *P*. *sp900767615* were mostly assigned to the available genomes of the corresponding species. To investigate functional differences between the two *Prevotella* species, we identified the differentially expressed genes (DEGs) between them (Table S3). Interestingly, we found that DEGs related to adaptive cellular responses pathways, e.g., *rcsC1*, *rcsC2*, *rcsC5*, *rcsC7*, were significantly up-regulated in *P*. *sp900767615* than *P*. *copri* and other Prevotella species (Fig. 3D). These up-regulated genes were sensor histidine kinases which regulated adaptive cellular responses to chemical or physical state of the environments. The results indicated the functional heterogeneity as adaptive responses existed among species in the *Prevotella* genus.

In the *Roseburia* genus, a total of 712 cells were identified as three *Roseburia* species, including *R*. *hominis* (36%), *R*. *intestinalis* (18%) and *R*. *sp900552665* (46%), which related to Cluster #6 and Cluster #9, separately (Fig. 3C). The *R. hominis* and *R. intestinalis* are familiar bacterial species in the human gut. To investigate the functional differences between the three *Roseburia* species, we identified DEGs among them (Table S4). The genes related to cell motility, e.g., *hag1*, were significantly up-regulated in *R*. *hominis* (Fig. 3D), which consistent with previous report about *R*. *hominis* being a flagellated gut anaerobic bacterium (Patterson et al., 2017). In addition, the DEGs related to adaptive-response sensory (e.g., *sasA3*) and multidrug resistance (e.g., *mdtC*) are highly expressed in *R*. *intestinalis*. In summary, the results indicated that smRandom-seq2 could sensitively capture functional heterogeneity among bacterial species in a complex microbial community.

### Intra-population adaptive strategy heterogeneity in the human gut microbiome

**Figure 4.**
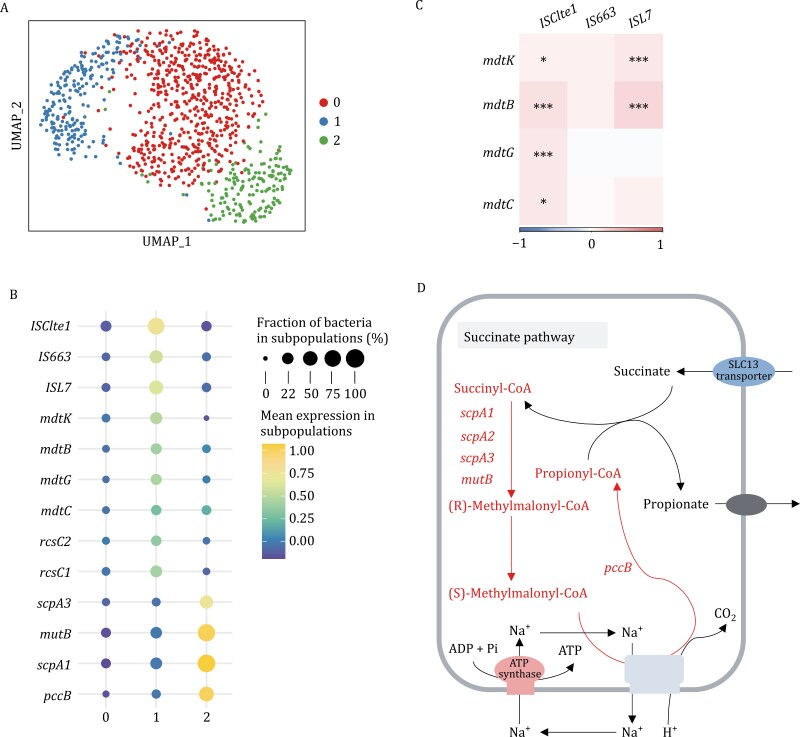
Functional heterogeneity in ***Phascolarctobacterium succinatutens*** in human gut microbiome. (A) UMAP plot shows three clusters (subpopulations) of *P*. *succinatutens* under 0.5 resolution of Seurat package. (B) Dot plot shows significantly up-regulated genes in the three subpopulations of *P*. *succinatutens.* (C) Gene expression level correlations between the mobile genetic elements related genes and multidrug resistance genes in subpopulation 1. (D) The genes in subpopulation 2 significantly up-regulated in succinate metabolism pathways in *P*. *succinatutens*. *ISClte1*:IS3 family transposase ISClte1. *IS663*: IS1182 family transposase IS663. *ISL7*: IS30 family transposase ISL7. *mdtK*: Multidrug resistance protein MdtK. *mdtG*: Multidrug resistance protein MdtG. *mdtB*: Multidrug resistance protein MdtB. *scpA*: Methylmalonyl-CoA mutase. *mutB*: Methylmalonyl-CoA mutase large subunit. *pccB*: Propionyl-CoA carboxylase beta chain.

Among all barcodes (cells) identified in the gut microbiome, *Phascolarctobacterium* is one of the most abundant genera. *Phascolarctobacterium* can produce short-chain fatty acids, including acetate and propionate, and associates with the metabolic state and mood of the host. Interestingly, all cells of the *Phascolarctobacterium* genus came from one species, *P*. *succinatutens*, which is an obligately anaerobic and gram-negative bacterium and colonizes the human gut (Ikeyama et al., 2020). We next analyzed the *P*. *succinatutens* smRNA data to understand whether it contained any intra-population functional heterogeneity in transcriptional states. In the gut microbiome, we identified three major subpopulations in both replicates using an unsupervised clustering approach (Fig. 4A) and further identified the DEGs among the three subpopulations (Fig. 4B; Table S5).

Among the cells from *P*. *succinatutens*, 22% of them fell into the subpopulation 1. Based on differential expression analysis, we found the DEGs related to mobile genetic elements (MGEs), e.g., *ISClte1*, *IS663*, and *ISL7*, which promote the evolution of antibiotic resistance, had significantly higher expression levels in the subpopulation (Figs. 4B and S5A). Interestingly, we indeed found that many multidrug resistance genes, e.g., *mdtB*, *mdtC*, *mdtG*, *mdtK*, and adaptive cellular responses related genes, e.g., *rcsC1*, *rcsC2*, were up-regulated in the subpopulation 1. The result provides a possible explanation for the subpopulation’s elevated multidrug resistance frequencies, and resistance likely is emerging from this subpopulation. To test this hypothesis, we performed a gene expression co-occurrence analysis between the MGE genes and multidrug resistance genes. Based on the analysis, we observed that there was a significant co-occurrence relationship between their expressions (Fig. 4C). We further measured the mutation frequencies in the subpopulation 1 of *P*. *succinatutens*. The results confirmed the hypothesis, i.e., the intrinsic functional heterogeneity driven by MGEs may promote the evolution of antibiotic resistance in *P*. *succinatutens*.

Based on DEG analysis, we found that genes in succinate pathway, e.g., *mutB*, *pccB*, were significantly high expressed in *P*. *succinatutens* in subpopulation 2 (Figs. 4B and S5B). Previous studies suggested that *P*. *succinatutens* uses succinate as a substrate rather than carbohydrates for growth in an energy-limited environment as one strategy to survive in the human gut (Ikeyama et al., 2020). Therefore we further investigated the expression level of the genes related to succinate metabolism pathways in *P*. *succinatutens* (Fig. 4D). Interestingly, among the genes involving the main steps of succinate metabolism, such as succinyl-CoA and methylmalonyl-CoA, most of them had significantly higher gene expression level in the subpopulation 2. The results suggested that the ability of chemical energy conversion via succinate was significantly increased in the subpopulation. Taken together, our smRandom-seq2 data revealed different adaptive strategies of asaccharolytic bacteria (e.g., *P*. *succinatutens)* in the human gut.

### Host-phage activity associations in the human gut microbiome

**Figure 5.**
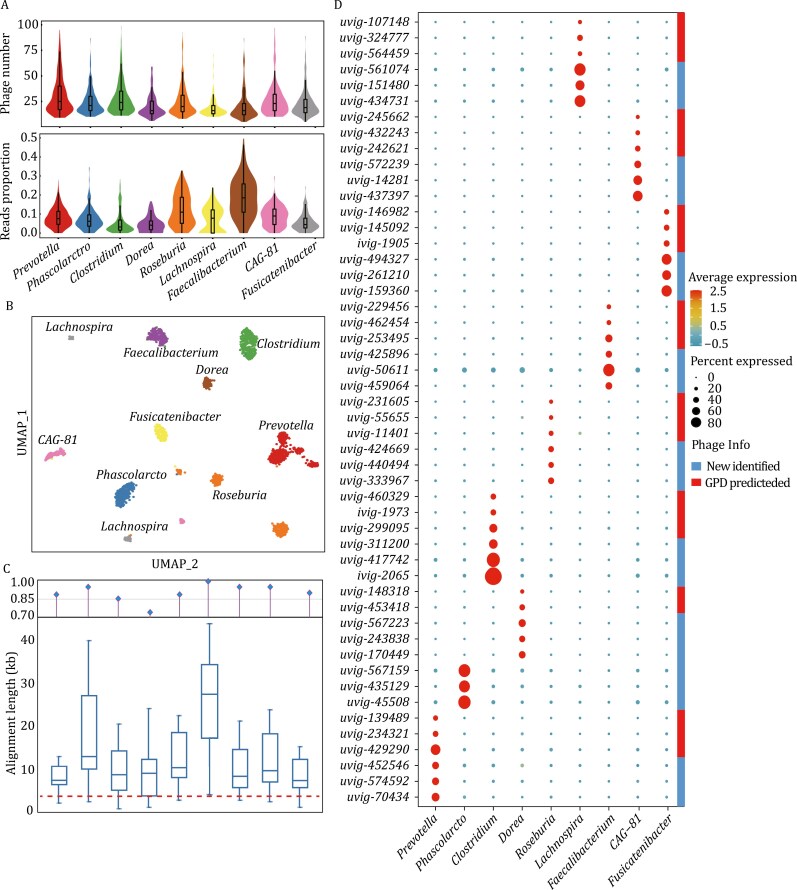
Bacterial host-phage transcriptional activity associations in different genera in the human gut microbiome. (A) Number of phages identified (up) and the proportion of reads aligned to the gut phage reference genomes in GPD (down) in bacterial cells. The data of nine main bacterial genera were shown. (B) UMAP plot of cells from the human gut microbiome based on both phage and bacteria smRandom-seq2 data with taxonomic annotation using MIC-Anno. (C) Alignment length distribution of top 20 phages identified in the nine genera based on the GPD reference genomes. An alignment length longer than 3 kb (red dashed line) is considered as an accurate prediction of host-phage relationship. Top chart indicated the prediction accuracy (%) of host-phage transcriptional associations. The rank of the nine genera is same as the above subfigure A. (D) Host-phage associations identified in the nine bacterial genera by this study. Beside of those same to the predicted ones by GPD, at least 325 associations were newly identified by this study.

Bacteriophages play extensive and important interactive regulatory roles in the human gut microbiome (Shkoporov et al., 2022). Given that smRandom-seq2 can simultaneously encapsulate bacteria as well as its associated phages, we further used MIC-Phage to investigate the host-phage interactive transcriptional relationships in the human gut microbiome at a single microbe level. We filtered out rRNA/tRNA sequences and then compared the smRandom-seq2 data to the Gut Phage Database (Camarillo-Guerrero et al., 2021) (GPD). We totally obtained 11.26% unique mapping reads of all the smRNAs in the human gut microbiome to the GPD database. Based on the taxonomic annotation results generated by MIC-Anno, we acquired a phage transcriptional profile from nine major genera in the human gut microbiome. The number of phages identified from per bacterium in each genus was 15 to 25. The ratio of phage-related sequences identified was different among the genera, from *Faecalibacterium* with the highest 19.2% phage-related sequences to the lowest 2.5% in the *Clostridium* (Fig. 5A). We clustered the bacterial cells using UMAP-based on transcriptional profiles of both phage and bacteria (Fig. 5B). Surprisingly, a very discriminative clustering result was achieved and the UMAP cell clusters matched extremely well with the nine major genera. Moreover, even it was further subdivided phage sequences from genus to species level, cell clustering, and taxonomic information still had an excellent consistence (Fig. S6).

According to the above clustering results, we further counted the phage-related UMIs to determine expression level information of main phage types in each genus. In order to verify the accuracy of the host-phage connectivity, we selected the top 20 phages with the most transcriptional activity in each genus (a total of 180 phages) identified by MIC-Phage. We estimated sequence similarity by mapping the phage to all reference genomes of the corresponding bacterial genus. As expected, all of the 180 phage genomes could be mapped into the reference genome with a threshold of alignment length for reliable relationship prediction (Fig. 5C). These results suggested that MIC-Phage could detect the host-phage activity associations in human gut microbiome accurately. In total, 373 reliable host-phage relationships were identified in this study (Fig. 5D). Among the host-phage relationships, at least 325 were newly identified by this study, and the rest 48 same as the predicted genus-level relationships by GPD (Table S6). Notably, the significant difference in expression level of phage-related genes among genera reflected the characteristics and ability of phage-specific infection (Figs. 5B and S6). Taken together, the results demonstrated the advantages of smRandom-seq2 to establish accurate *in vivo* host-phage activity connectivity.

### Application of smRandom-seq2 on three more human gut microbiomes

**Figure 6.**
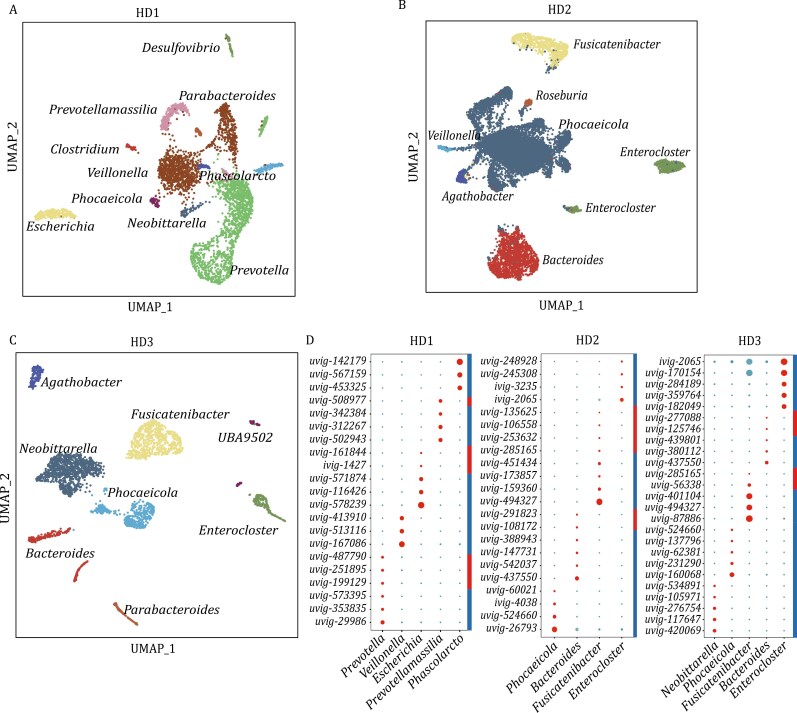
Single-microbe transcriptional landscape and host-phage associations of the gut microbiome in more healthy donors. (A–D) UMAP plot of smRandom-seq2 data clusters (A–C) and dot plot of host-phage associations (D) in three more healthy donors (HD1, HD2, HD3).

To further validate and extend the generalizability of smRandom-seq2 in real-world situations, we went on to perform smRNA-seq in three fecal samples from healthy human subjects. In total, 21,264 cells were captured from the three samples, with an average depth of 13,380 reads per cell. Based on the annotation of MIC-Anno, the three healthy donors had distinct dominant bacteria genus/species in their guts, *Prevotella*/*P*. *copri* in one donor, *Neobittarella*/*N*. *massiliensis* in one donor, and *Phocaeicola*/*P*. *coprocola* in another one donor, respectively (Fig. 6A–C). The UMAP cell clusters based on the gene expression profiles of the samples matched well with the bacteria genus and species annotated above (Fig. 6A–C). Subpopulations could be identified in some dominant species (*Prevotella copri* and *Phocaeicola dorei*, etc.) (Fig. 6A and 6B). Meanwhile, we totally identified 256 reliable host-phage activity relationships from the healthy donors using MIC-phage (Fig. 6D). All these results suggested that smRandom-seq2 could adapt to various human gut microbiomes with distinct microbe communities and efficiently capture different bacteria species in real-world situations.

## Discussion

Several bacterial scRNA-seq methods have been developed recently, including plate-based techniques like microSPLiT (Kuchina et al., 2021), PETRI-seq (Blattman et al., 2020), MATQ-seq (Imdahl et al., 2020) and probe-based methods like par-seqFISH (Dar et al., 2021), ProBac-seq (McNulty et al., 2023), and droplet-based methods like BacDrop (Ma et al., 2023), smRandom-seq (Xu et al., 2023a). These developments are timely and important. For instance, Kuchina et al. developed microSPLiT and applied it to *Bacillus subtilis* sampled at different growth stages, and identified the heterogeneous activation of a niche metabolic pathway (Kuchina et al., 2021). Ma et al. applied BacDrop to study *K. pneumoniae* clinical isolates and to elucidate their heterogeneous responses to antibiotic stress (Ma et al., 2023). Dar et al. applied par-seqFISH to the opportunistic pathogen *Pseudomonas aeruginosa* across dozens of conditions in planktonic and biofilm cultures and identified numerous metabolic- and virulence-related transcriptional states that emerged dynamically during planktonic growth (Dar et al., 2021).

The currently available techniques however are usually focused on single well-characterized bacteria species, which are not adapted to human microbiome research because of the complexity of natural microbial communities. There are several critical challenges when it comes to clinical- and physiology-relevant human complex microbiome samples: (i) The composition of human microbial community is much more complicated than cultured bacteria, which causes the capture efficiency of common random primers to significantly decrease, leads to the bacteria capture bias and results in a low number of species and genes detected in the microbial community samples. (ii) Compared with cultured bacteria, microbial community samples are more prone to bacterial aggregation leading to serious cross-contamination in the microbial community sequencing. (iii) The digestion of different bacterial cell walls in microbial community samples leads to the presence of bias, which can result in fewer detected bacterial species. (iv) The human gut microbiome samples contain huge amounts of impurities that will interfere with the experiment processes and reactions. (v) Bioinformatics tools are not available for dealing with scRNA data from a complex microbiome. There are many challenges to identifying single microbe and bacteria(host)-phase associations based on the limited RNA reads.

In our latest study, we developed a droplet-based high-throughput and high-sensitivity smRNA-seq method (smRandom-seq2) for complex microbial communities. As compared to the existing smRNA-seq methods, smRandom-seq2 overcomes the challenges of high species diversity and heterogeneity that are ubiquitously present in real-world microbiome samples. The key behind smRandom-seq2’s advances is: (i) In smRandom-seq2, the novel design of random primers significantly increases the efficiency of reverse transcription for all bacteria species. We have screened hundreds of different random and semi-random primer designs, and a set of GAT random primers with the highest efficiency was ultimately selected. (ii) In smRandom-seq2, the pre-index-based experiment process significantly reduces cross-contamination in the single microbe sequencing of complex microbial communities. We introduced the pre-index design on the GAT random primers to reduce the cross-contamination caused by bacterial aggregation. Based on the pre-index strategy, we can distinguish two bacteria even if they were included in the same droplet. Our results showed that the mean species purity of barcodes is higher than 95% in smRandom-seq2, which is very hard to achieve by other methods on complex microbiomes. (iii) Previous single microbe sequencing platforms, like BacDrop and other droplet-based single bacterial sequencing technologies, are developed based on the 10× Genomics single cell barcoding platform, which were primarily developed for eukaryote (like human or mouse) single cells and were not efficient for single microbe barcoding due to the much smaller size of microbes and huge amount of impurities existed in microbiome samples. In smRandom-seq2, we developed a high barcoding efficiency droplet platform specifically suitable for the complex microbiome single microbe sequencing. (iv) In smRandom-seq2, we also developed a novel computational pipeline for single microbe analysis in complex microbial communities. This computational method for the first time solved the problem of multiple species single cell analysis, and is extremely suitable for single microbe sequencing in complex microbial communities. By pooling the smRNA-seq data by smRandom-seq2, we are able to obtain accurate taxonomic information for each microbe and further bacterial and host-phage association analysis. Due to the above improvements in this study, we were able to make it possible for single microbe sequencing in human gut microbiome samples. Compared to the existing single microbe RNA sequencing methods, such as microSPLiT (median UMI: 235, median gene: 138), PETRI (median UMI: 227, median operon: 103), MATQ-seq (mean gene: 170), BacDrop (mean gene: 90), the UMI and the gene number per cell of smRandom-seq2 (median UMI: 1,158, median gene: 246) is superior (Fig. S7).

Indeed, the applications of smRandom-seq2 on human fecal samples demonstrated that smRandom-seq2 could sensitively dissect functional heterogeneity existing in complex microbial communities and identify host-phage activity relationships at the genus and species level. smRandom-seq2 can also simultaneously identify hundreds of bacterial species (many poorly known) from the human gut, highlighting its unique advantages in the depth and scope of species coverage, and also its efficiency. By applying smRandom-seq2 in human fecal samples, we have generated tens of thousands of human single microbes that the research community can readily access and utilize. Together, our results support that smRandom-seq2 has the potential for a variety of biomedical and even clinical research, from understanding the microbiome functional changes, and bacteria dynamic communications, to bacteria metabolic networks and host-microbiome interactions and so on. We also aim smRandom-seq2 to be highly user-friendly and cost-effective, enabling many labs and hospitals to rapidly adapt smRandom-seq2 for their research and discovery needs, or even clinical management needs in the future. Furthermore, the ability for smRandom-seq2 to capture microbiome transcriptional status in single microbe resolution promises new avenues for the understanding of complex microbial communities and their functional impact on human health.

Based on the systematic phage analysis in single microbes, we found majority of the phages were related to actively transcribed prophages. After further investigations, we found a vast majority of these phage-aligned reads (over 85%) can be mapped to bacterial genomes. This indicates that the majority of viruses are at prophage status, and the transcription activity of prophages was significantly related to the adaptive states of the bacteria in the human gut microbiome. Among the 373 reliable predicted host-phage relationships we identified, each phage species is essentially associated with a specific bacterial genus. Only 8 bacteriophages exhibit associations with multiple bacterial genera. Many of these phages among them are uncultivable, thus a substantial portion lacks taxonomic and genomic information in the dataset (only 16,636 out of 142,809 phages possess family-level information). Among the 373 host-phage relationships we identified in the gut microbiome sample, we identified 18 relationships involving hosts and phages with clearly identified taxonomic information. These phages primarily belong to Caudovirales order and one Microviridae family bacteriophage. These 18 relationships are all consistent with the current research findings and predictive results (Fujimoto et al., 2021; Manrique et al., 2016; Mayneris-Perxachs et al., 2022). In addition, we uncovered that these prophage-related bacterial functional genes primarily engage in pivotal functional pathways, notably arginine and tryptophan metabolism. Remarkably, these findings align with the research documented by Fujimoto et al. (Fujimoto et al., 2021). Additionally, some functional genes such as htpG exhibit a correlation with bacterial stress protection, suggesting their potential role in enhancing the coexistence of bacteria with prophages that support resilience.

There are several potential avenues for further improvement and expansion of our technique. First, although smRandom-seq2 could capture thousands of single microbes in microbiome samples, the throughput is still far from the real bacteria number in natural microbial communities. The design with more barcodes and smaller droplets has the potential to substantially increase the throughput of this method. Second, the development of a universal rRNA depletion method would avoid the sequencing of non-messenger transcripts, thus reducing the cost of sequencing. In smRandom-seq2, we detected a ribosomal RNA (rRNA) proportion ranging from 75% to 95% among the samples. Even without rRNA depletion, we observed a higher number of genes compared to existing single-bacteria RNA sequencing methods, and we have not yet reached saturation which suggested more genes could be captured when the sequencing depth increased (Fig. S8A). Thirdly, it is possible that the different clusters within the same taxonomic group represent different strains in the same species. The reason is that the single microbe annotation method (MIC-Anno) could only classify the barcode into species taxon level. So the different clusters could represent the transcriptional/functional differences between the clusters in one species, which could be both the same or different strains. Fourthly, exploration of additional capture and separation methods for both host and microbe cells could enable dissecting the transcriptional activity in both human cells and single microbes in the same sample, leading to a deep understanding of the host-microbe interactions in the human gut. Furthermore, combining smRandom-seq2 with other multi-omics techniques, such as proteomics and metabolomics, will able to capture the functional changes of single microbes in multiple layers, and significantly advance our understanding of the complex interplay between the microbes and host in human health and disease.

## Materials and methods

### Bacterial culture and collection

The bacteria used for the experiment are *E. coli* BW25113 (*E*. *coli*), *A. baumannii* ATCC17978 (*A*. *baumannii*), *K. pneumoniae* XH209 (*K*. *pneumoniae*), *Pseudomonas aeruginosa* PAO1 (*P*. *aeruginosa*) and *S. aureus* subsp. Aureus SA268 (*S*. *aureus*), which were obtained from Sir Run Run Shaw Hospital, Zhejiang University School of Medicine. The bacteria were cultured overnight in LB liquid medium (Sigma Aldrich, L3522) at 37°C with shaking (250 rpm). Based on the purpose of different experiments, cultures of different bacterial stains were sampled upon reaching the OD_600_ ~0.2, and immediately centrifuged at 4°C, 6,000 ×*g* for 2 min, next washed twice by PBS, and mixed before applied with smRandom-seq2.

### Human fecal sample collection

The fecal samples were collected from four healthy donors. The study protocol was approved by the Ethics Committee of the First Affiliated Hospital, Zhejiang University School of Medicine, China (2021IIT A0239). All the participants provided written informed consent. The samples were centrifuged twice at 4°C, 500 ×*g* for 3 min to eliminate impurities from the digested food or host cells. The obtained samples were then centrifuged at 4°C, 3,900 ×*g* for 5 min to collect the bacteria, ensuring high purity. The bacteria collected were used for further smRandom-seq2 or stored at −80°C until needed for additional metagenomic sequencing.

### Fixation and permeabilization

The bacteria were first fixed with 4% paraformaldehyde (PFA) at 4°C overnight, which helps to preserve their structure and prevent degradation. Next, the cells were washed and incubated with 0.04% Tween-20 in PBS. This detergent helps to create small pores or holes in the bacterial cell membrane, allowing other molecules like enzymes to enter the cell. Then, the cell wall was permeabilized with lysozyme (2.5 mg/mL, ThermoFisher, 90082) and lysostaphin (0.0125 mg/mL, Sigma, L7386). Lysozyme is an enzyme that breaks down the peptidoglycan layer in bacterial cell walls, while lysostaphin is an enzyme that specifically targets and breaks down the cell walls of *Staphylococcus* species. After the digestion step, the bacteria were immediately washed and resuspended in PBS with RNase inhibitor (ThermoFisher, Cat#AM2694), which helps to protect any RNA present in the sample from degradation by RNases. For the cell loss problem, we conducted a comprehensive evaluation of various centrifugation protocols to mitigate cell loss. We explored a range of centrifuge types, including those with horizontal and angled rotors, varied capacities of centrifuge tubes, and diverse centrifugation reagents. After a systematic optimization, we identified that employing a centrifuge with a horizontal rotor, using 200 μL EP tubes, and resuspending cells in 0.05% PBST (Phosphate-Buffered Saline with Tween 20) prior to centrifugation yielded the most favorable results. Upon completing the experimental workflow, we were able to retain over 40% of the initial bacterial population, as shown in Fig. S8B.

### Reaction *in situ*

*In situ* reactions of bacteria were carried out using a commercially available reverse transcription kit from M20 Genomics (R20114124). The kit contained reverse transcriptase (50 U/µL), 5× reverse transcription buffer, and dNTP Mix (100 mmol/L). A 50-µL reaction mixture was prepared, consisting of about 5 million bacteria in 27.5 µL DEPC water, 10 µL 5× reverse transcription buffer, 5 µL 10 µmol/L random primer (Table S5), 2.5 µL 100 mmol/L dNTP, 2.5 µL RNase inhibitor, and 2.5 µL reverse transcriptase (50 U/µL), and subjected to ten cycles of multiple annealing ramping from 8°C to 42°C, followed by a 42°C incubation for 30 min in a thermal cycler. After the RT reaction, bacteria were washed five times with 0.05% PBST. The bacteria were then subjected to dA-tailing by adding them to 39 µL DEPC water, followed by the addition of 5 µL 10× TdT buffer, 5 µL 2.5 mmol/L CoCl_2_, 0.5 µL 100 mmol/L dATP, and 0.5 µL TdT enzyme, and incubated at 37°C for 30 min. The bacteria were subsequently washed with PBST three times.

### Microfluidic device fabrication

Microfluidic devices based on PDMS were designed and fabricated for the synthesis of hydrogel beads, following a previously described protocol (Wang et al., 2020). The channel depth of the microfluidic devices used for hydrogel bead synthesis was 30 μm, while the devices used for cell encapsulation had a channel depth of 50 μm. To create molds for the microfluidic devices, a photolithographic method was employed, involving centrifugal coating and modeling of SU-8. Subsequently, PDMS (Sylgard-184) was cast onto the silicon molds to fabricate the microfluidic devices.

### Microfluidic platform

A microfluidic platform for barcoding of single-bacteria was established based on previous work (Wang et al., 2020). The platform equipment comprises display monitors, a pressure controller, a microfluidic chip, and an inverted bright-field microscope equipped with a high-speed camera and a computer for data acquisition and analysis.

### Barcoded beads synthesis

Hydrogel barcoded beads for single bacterium barcoding were developed based on previous studies and were customized by M20 Genomics company (Ko et al., 2021; Wang et al., 2020). These hydrogel beads were synthesized using microfluidic emulsification and polymerization of an acrylamide-primer mix that includes acrylamide:bis-acrylamide solution, acrydite-modified oligonucleotides, ammonium persulfate, and Tris-buffered saline-EDTA-Triton buffer. The acrydite-modified oligonucleotides contain a deoxy Uridine base, replacing the photocleavable moiety present in previous reports. A carrier oil-TEMED mix was applied in microfluidic to facilitate synthesis. The acrylamide-primer mix and carrier oil-TEMED mix were transferred to syringes, respectively, and connected to corresponding inlets of the hydrogel bead synthesis device. The generated hydrogel beads should have a size of 40 μm. The DNA primers on the hydrogel beads were barcoded using a combination of a split-and-pool method and a 3-step primer ligation reaction. Unique barcoded primers were used, and they are provided in Table S6. Hydrogel bead mix, DNA ligase, dNTP, isothermal amplification buffer, and nuclease-free water were prepared and split into a round-bottom 96-well plate. The hydrogel bead mix in the 96-well plate was mixed with 96 annealed unique barcode primers in another 96-well plate, respectively, and then incubated. All hydrogel beads were combined in a single tube and washed, and the second and third split-and-pool rounds were performed. To verify the quality of the generated hydrogel barcoded beads, barcoded primers were released by Uracil-specific excision reagent enzymatic digestion and analyzed with gel electrophoresis. The highest molecular-weight peak represents the full-length barcoding primer, and lower-molecular-weight peaks are synthesis intermediates. All the necessary reagents for hydrogel barcoded bead synthesis and the ready-to-use hydrogel barcoded beads can be ordered from M20 Genomics company.

### Droplet barcoding

To perform the modified droplet barcoding for a single bacterium, qualified individual bacteria were first counted under an optical microscope and then diluted with a 15% density gradient solution. Before droplet generation, we quantified the bacterial count through microscopy, as depicted below. We then appropriately diluted the sample to achieve an optimal concentration for the microfluidic loading procedure. We optimized the loading approach to achieve approximately 30% of droplets containing bacteria, as opposed to the traditional loading strategy where only 10% of droplets contained cells. This method ensures that the majority of bacteria are enclosed within individual droplets. Additionally, the overloaded bacteria can be distinguished through the pre-index sequence. The microfluidic platform was used to encapsulate bacteria, 2× DNA extension reaction mix, and hydrogel barcoded beads. Both the density gradient solution and 2× DNA extension reaction mix were obtained from M20 Genomics. The collected droplets were subjected to a series of incubation steps at different temperatures: 37°C for 1 h, 50°C for 30 min, 60°C for 30 min, and 75°C for 20 min. The droplets were then broken by mixing with PFO buffer, and the oil phase was discarded. The aqueous phase containing cDNAs was purified using Ampure XP beads. We achieved a promising 76% success rate in generating unique barcodes for individual bacterial cDNAs, and only 2% of droplets containing two or more beads. After the loading process, about 55% of the beads were *without bacteria*, and around 0.6% of the bacteria remained without any beads.

### cDNA enrichment

After cDNA purification, a qPCR reaction was performed to determine the cycle numbers required for cDNA enrichment. The cycle numbers at which the qPCR reaction reached the early exponential amplification phase were identified as the cycle numbers for cDNA enrichment. Subsequently, PCR amplification was carried out using specific primer sets (Table S1). The resulting PCR products were purified again using Ampure XP beads, and the purified cDNAs were quantified using Qubit.

### Library preparation

For library preparation, we used the VAHTS Universal DNA Library Prep Kit for Illumina V3 (ND607-03/04 Vazyme). The amplified and purified cDNAs were quantified using a Qubit2.0 and analyzed using the Qsep100™ DNA Fragment Analyzer. End-repair and adenylation were performed on the qualified cDNAs using a reaction mixture containing 50 ng fragmented DNA, end-repair enzymes, end-repair buffer, and nuclease-free water. The reaction mixture was incubated at 30°C for 30 min and then inactivated at 65°C for 30 min. After adding the working adaptor and ligation enzymes, the mixture was incubated at 20°C for 15 min, and the ligated DNA was then purified and selected using AMPure XP beads. The library was then amplified by PCR and purified again using AMPure XP beads. Finally, the qualified cDNA library was quantified using a Qubit2.0 and analyzed using the Qsep100™ DNA Fragment Analyzer, before being sequenced using the NovaSeq 6000 and S4 Reagent Kit with paired-end reads of 150.

### Library generation and analysis for metagenome sequencing

DNA was extracted from frozen fecal samples using a TIANamp Micro DNA kit (DP316, Tiangen Biotech) according to the manufacturer’s recommendations. Agilent 4200 TapeStation (Agilent Technologies) was used to assess DNA quality. DNA libraries were carried out by a standardized procedure for DNA fragmentation, end repair, adapter ligation, and PCR amplification. Agilent Bioanalyzer 2100 was used to assess library quality. Whole-genome shotgun sequencing of fecal samples was performed on an Illumina Hiseq2500 platform. All samples were paired-end sequenced with a 150-bp read length to a targeted data size of 5.0 Gb. Metagenome data process: Sequence reads were passed through the KneadData QC pipeline, which incorporates the Trimmomatic and BMTagger filtering and decontamination algorithms to remove low-quality read bases and reads of human origin, respectively: (i) trim non-human reads shorter than 50 nucleotides; (ii) exclude samples with < 500,000 microbial reads. Taxonomic profiling was performed using the MetaPhlAn2 classifier. The classifier relied on approximately 1 million clade-specific marker genes derived from > 10,000 microbial genomes to unambiguously classify reads to taxonomies and yield the relative abundances of the taxa identified in the sample.

### Single microbe analysis pipeline for microbial community

Single microbe annotation algorithm (MIC-Anno): We first determined the cut-off values by the analysis of barcode and gene count scatter plots. We identified a point of inflection in the scatter plot that helped us select cells for further analysis. The portion of genes associated with cells located beyond this inflection point was automatically filtered out. In this method, we adopted a *K*-mer-based root to leaf taxonomic classification strategy. We first applied Kraken2 (Lu et al., 2022), a *K*-mer-based reads classification method, on every read in each barcode based on UHGG (v2.0.1) (Almeida et al., 2021) gut microbiome genome database. After all the reads were assigned to each node of different taxonomic levels (i.e., order, family, genus, and species), we calculated the sum of reads in each node from the leaf to the root. Then we performed the taxonomic classification from the root to leaf taxonomic levels. At the root taxonomic level, we ranked all the nodes from the highest to lowest based on the read number of the nodes and selected the node with most read number as a potential annotation candidate. Based on the annotation result, then we performed the same annotation process in the next lower taxonomic level, until the leaf nodes (species level). To evaluate the significance of the results, we also calculate the *P*-value for the prediction results of each node. We tested the MIC-Anno method in the mock community, and the results showed that it could precisely annotate each barcode.

Single microbe bacteria analysis pipeline (MIC-Bac): In the single microbe sequencing dataset, we identified hundreds of bacteria species in each microbiome sample, and some species could only detect few barcodes. So to further investigate the functional heterogeneity in species, we only keep the abundant bacteria species (> 3% total barcodes) for the downstream analysis. And it will be optional for the users to include all the genera in the analysis. First, we trimmed primer sequences and extra bases generated by the dA-tailing step in raw pair-end sequencing data. Then 8 bp UMI and 20 bp cell-specific barcode were extracted from R1 end sequencing file and merged as the same accepted barcode with a Hamming distance of 2 bp or less. And R2 end file was used to generate the bacterial gene expression matrix by STAR (v2.7.10a) (Dobin et al., 2013), featureCounts (v2.0.3) (Liao et al., 2014) and umi_tools (v1.1.2) (Smith et al., 2017) with reasonable parameters and whole UHGG (v2.0.1) (Almeida et al., 2021) gut microbiome genome as the reference. The high-quality and unique mapped reads were preserved to count UMIs for each barcode. Given that excess taxonomic types may produce noise to downstream analysis, we preserved a rational amount of major genera for the sample based on the species annotation result of MIC-Anno. Then we utilized dimensional reduction of single-cell expression vectors, followed by graph-based clustering and analysis of differentially expressed genes using the Seurat (v4) (Hao et al., 2021) package and a two-sided Wilcoxon rank-sum test with Bonferroni correction to identify unique cellular transcriptional differences (Kopylova et al., 2012). To detect the SNPs in each subpopulation, we first combined all the sequencing reads of each barcode in one subpopulation, and then used the software Bowtie2 to map the reads to the reference genome of the corresponding bacteria species, and used the software GATK to call the SNPs in each subpopulation.

Single microbe phage analysis pipeline (MIC-Phage): To contrast a transcriptional correlated matrix of phage and bacteria, we first built a phage-related gtf file for GPD genome sequences, which was a part of the input of STAR (v2.7.10a) (Dobin et al., 2013). Second, we filtered out the vast majority of rRNA and tRNA reads by SortMeRNA (Kopylova et al., 2012) for the quality-controlled data. Then we used STAR to align reads and the unique map reads were counted by featureCounts (v2.0.3) (Liao et al., 2014). Finally, we utilized umi_tools (v1.1.2) (Smith et al., 2017) to count UMIs and build the matrix. We used Seurat with reasonable parameters and removed the barcode with taxonomic information which bacteria belonging to this genus fewer than 3% in this sample further clustering and running dimensional reduction for visualization. Genus-related phages were identified following the steps of Seurat FindMarkers function and the logFC.threshold was set 0.1. For the top 20 phages of each genus we used blastn to compare the reference viral genome to UHGG (v2.0.1) genus reference genome. The solid prediction of host-phage correlation must have at least 3 kb alignment length and above 80% idents of both genomes.

## Supplementary data

The online version contains supplementary material available at https://doi.org/10.1093/procel/pwae027.

pwae027_suppl_Supplementary_Material

pwae027_suppl_Supplementary_Tables

## Data Availability

The smRandom-seq2 single-microbe RNA-seq datasets produced in this research are available in Genome Sequence Archive under the BioProject accession code “PRJCA017256”. The source data of the experiment is supplied in this paper.

## Code availability

The code of smRandom-seq2 analysis pipeline and further analysis for this paper has been presented on Github.
