# Supplementary material for: High-throughput single-microbe RNA sequencing reveals adaptive state heterogeneity and host-phage activity associations in human gut microbiome
Source: Protein Cell. 2024 May 23;16(3):211–26. doi: 10.1093/procel/pwae027 (PMC11891138; doi:10.1093/procel/pwae027)
Supplement: pwae027_suppl_Supplementary_Material [file pwae027_suppl_supplementary_material.pdf]

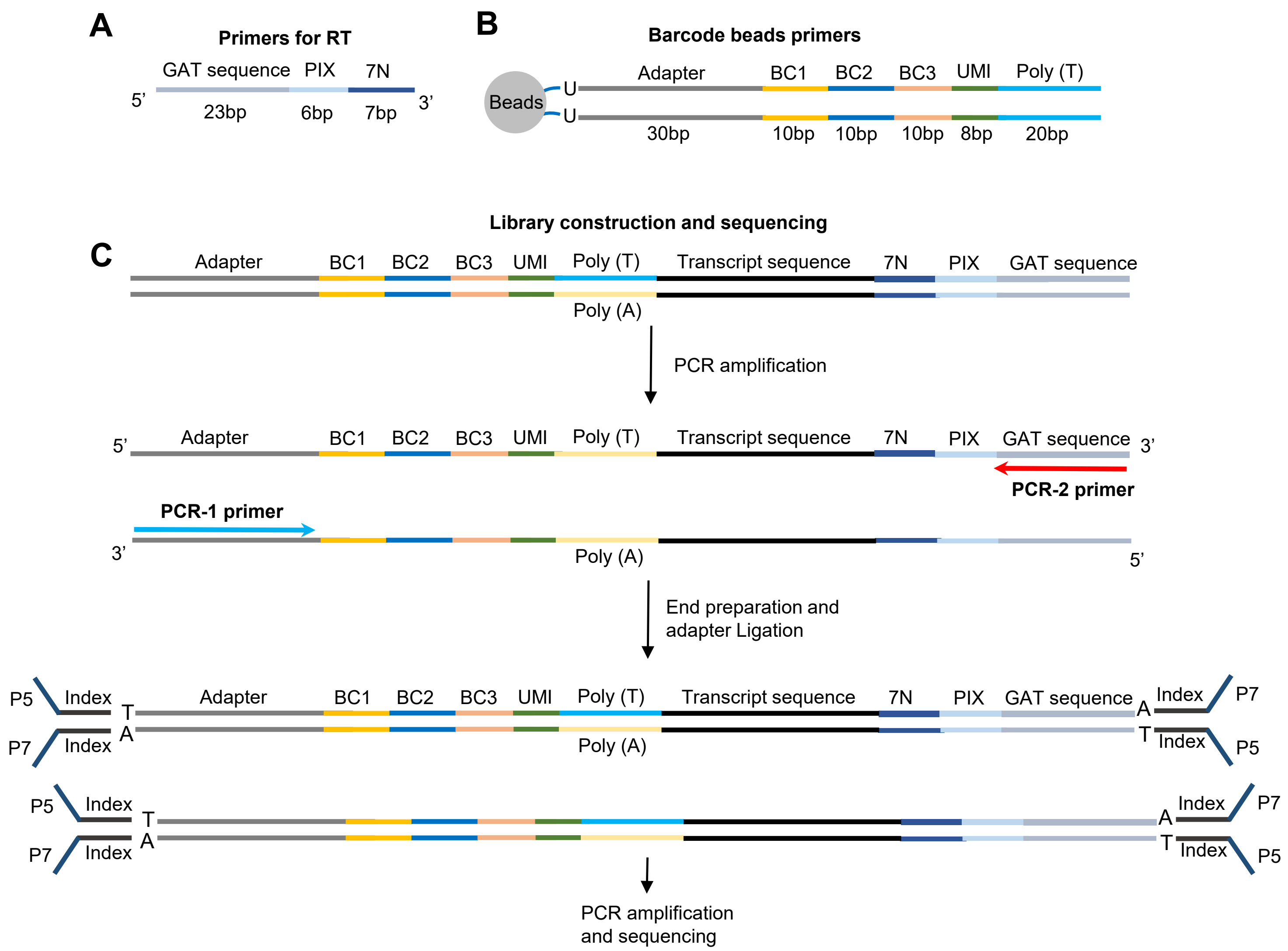

Figure S1. Illustration of smRandom-seq2 library preparation and sequencing strategy. (A, B) The design of RT and bead primers. PIX: Pre-index sequence that for distinguishing the bacteria with one barcode. BC: Barcode. UMI: Unique molecular identifiers. (C) smRandom-seq2 sequencing library preparation scheme for Illumina platforms.

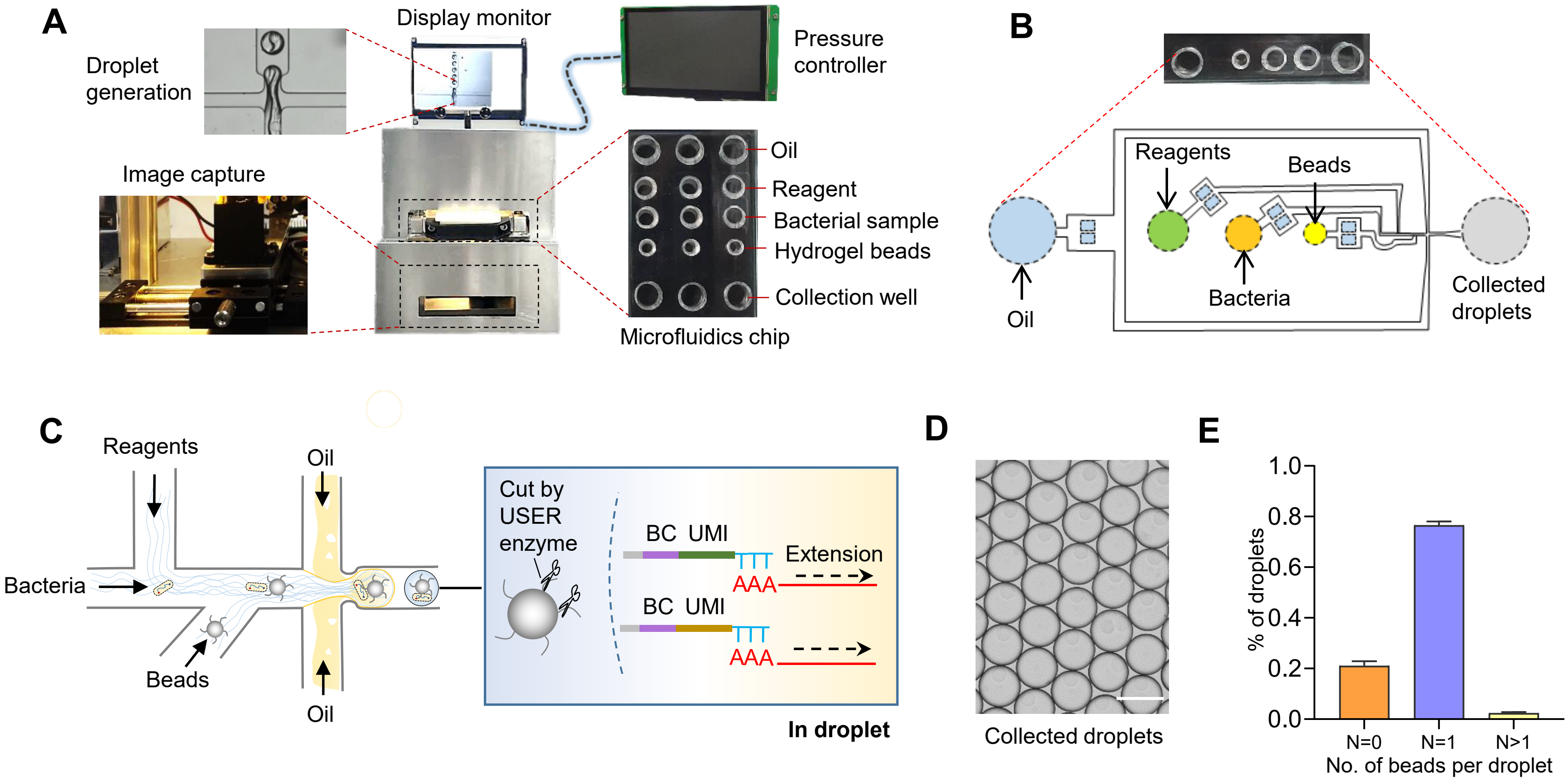

Figure S2. Automated droplet barcoding system for microbes. (A) Automated droplet collection system. (B) Design of the microfluidics chip for droplets generation. (C) Second-strand cDNAs synthesis in droplet. BC: barcodes. (D) Image of encapsulated droplets. Scale bar: 100 µm. (E) Percentage of droplets containing 0 gel bead ($N = 0$), 1 gel bead ($N = 1$) and >1 gel bead ($N > 1$). Data include five independent runs from multiple chip, $N = 5$, mean±s.e.m.

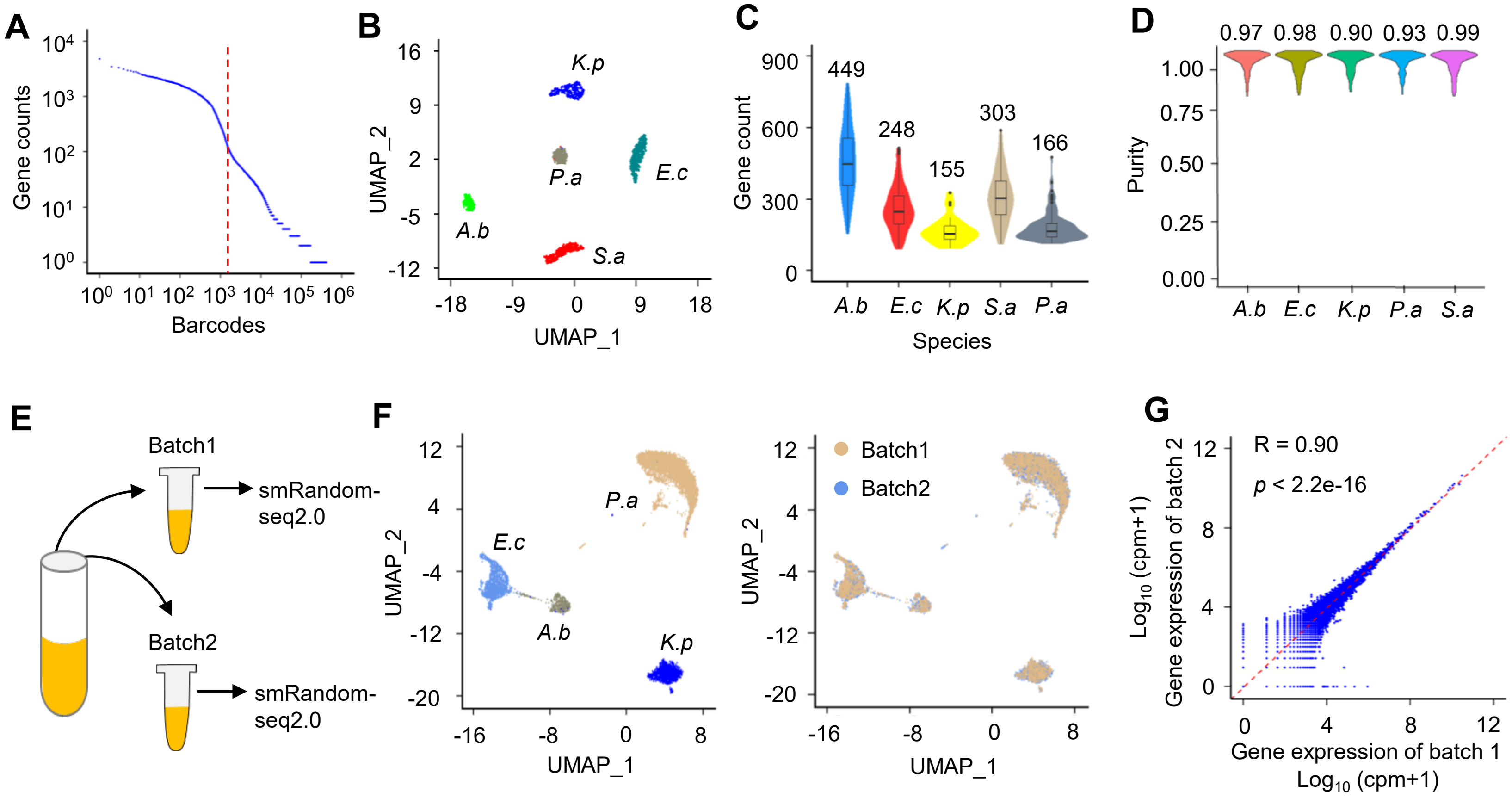

Figure S3. Validation and technical performance of smRandom-seq2. (A) Gene count versus barcode rank plot. (B) UMAP plot of the smRandom-seq2 cells and clusters colored by species. (C) Distribution of gene count of the five bacterial species clusters by smRandom-seq2. The median gene count of each species is presented at the top of the violin plot. The mock community includes *A. baumannii* (*A. b*), *E. coli* (*E. c*), *K. pneumonia* (*K. p*), *P. aeruginosa* (*P. a*), and *S. aureus* (*S. a*). (D) Violin plot shows the purity of each genus. The median purity of each species is presented at the top of the violin plot. (E) Experimental design for batch effect. (F) UMAP projection of two repeated bacteria mixture samples (Batch1 and Batch2) applied with smRandom-seq2, separately. The left image grouped by different clusters, while the right picture clustered by various groups. (G) Scatter plot showing correlation of gene expression (Log_{10}(cpm+1)) for two repeated for bacteria mixture samples (Batch1 and Batch2) applied with smRandom-seq2 separately.

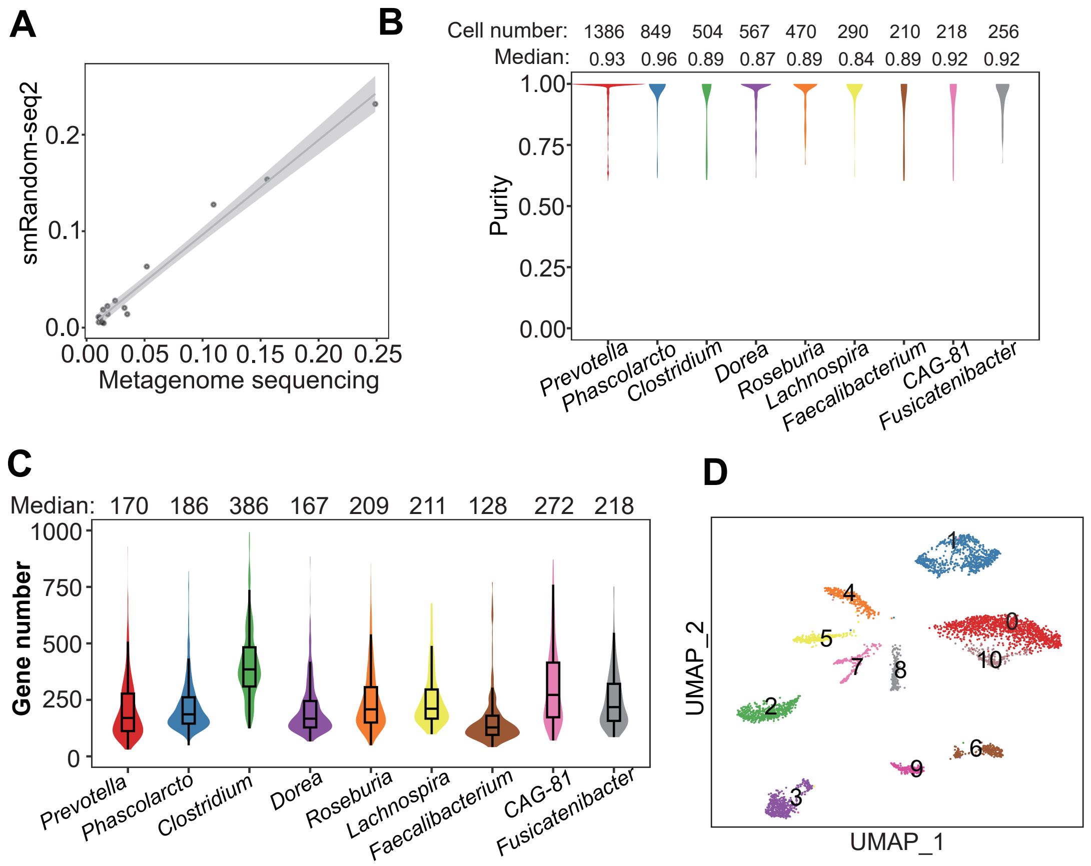

Figure S4. Single bacterial gene expression landscape in human gut microbiome. (A) Bacterial abundance correlation between smRandom-seq2 and metagenome sequencing. (B) Violin plot shows the purity of each genus. (C) Violin plot shows gene number of each genus. (D) UMAP plot shows clusters learned in Seurat package under the resolution of 0.5.

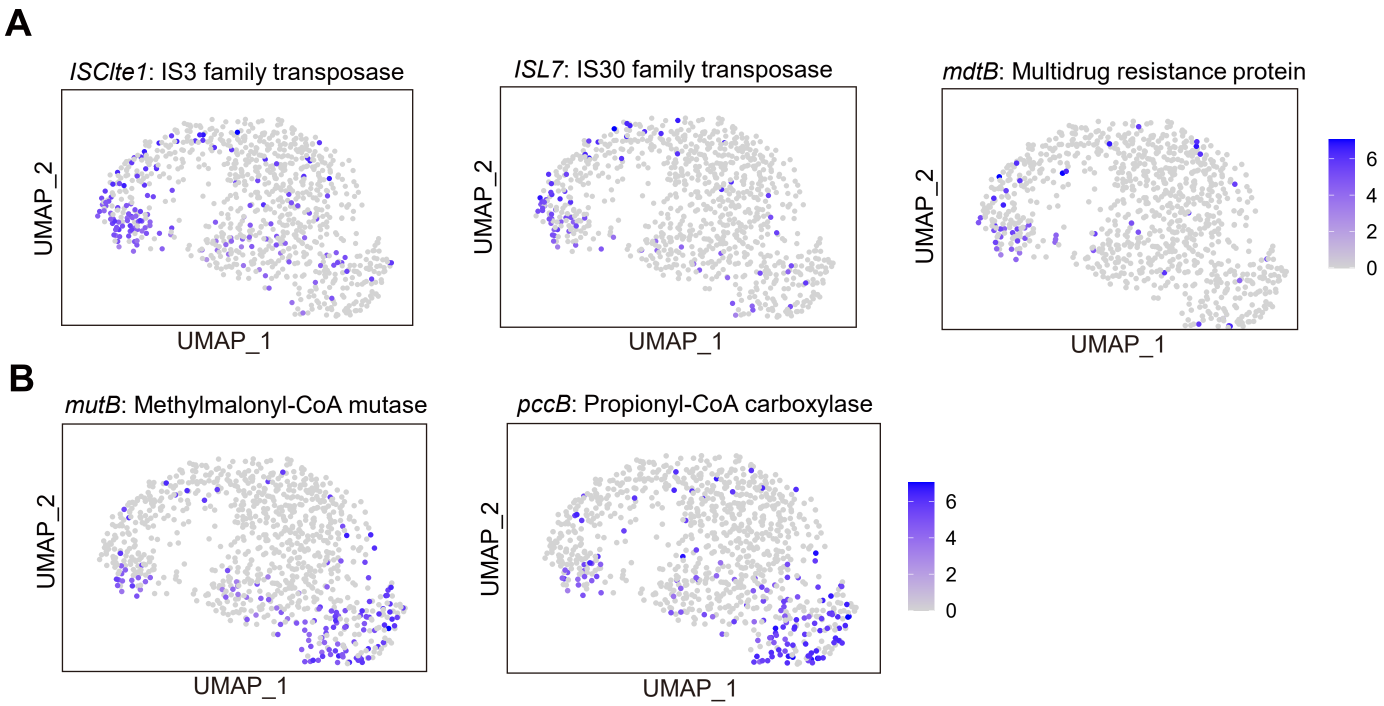

Figure S5. High expression gene in subpopulation of *Phascolarctobacterium succinatutens*. (A) UMAP plot shows higher expression gene in each barcode of subpopulation 1. (B) UMAP plot shows higher expression gene in each barcode of subpopulation 2.

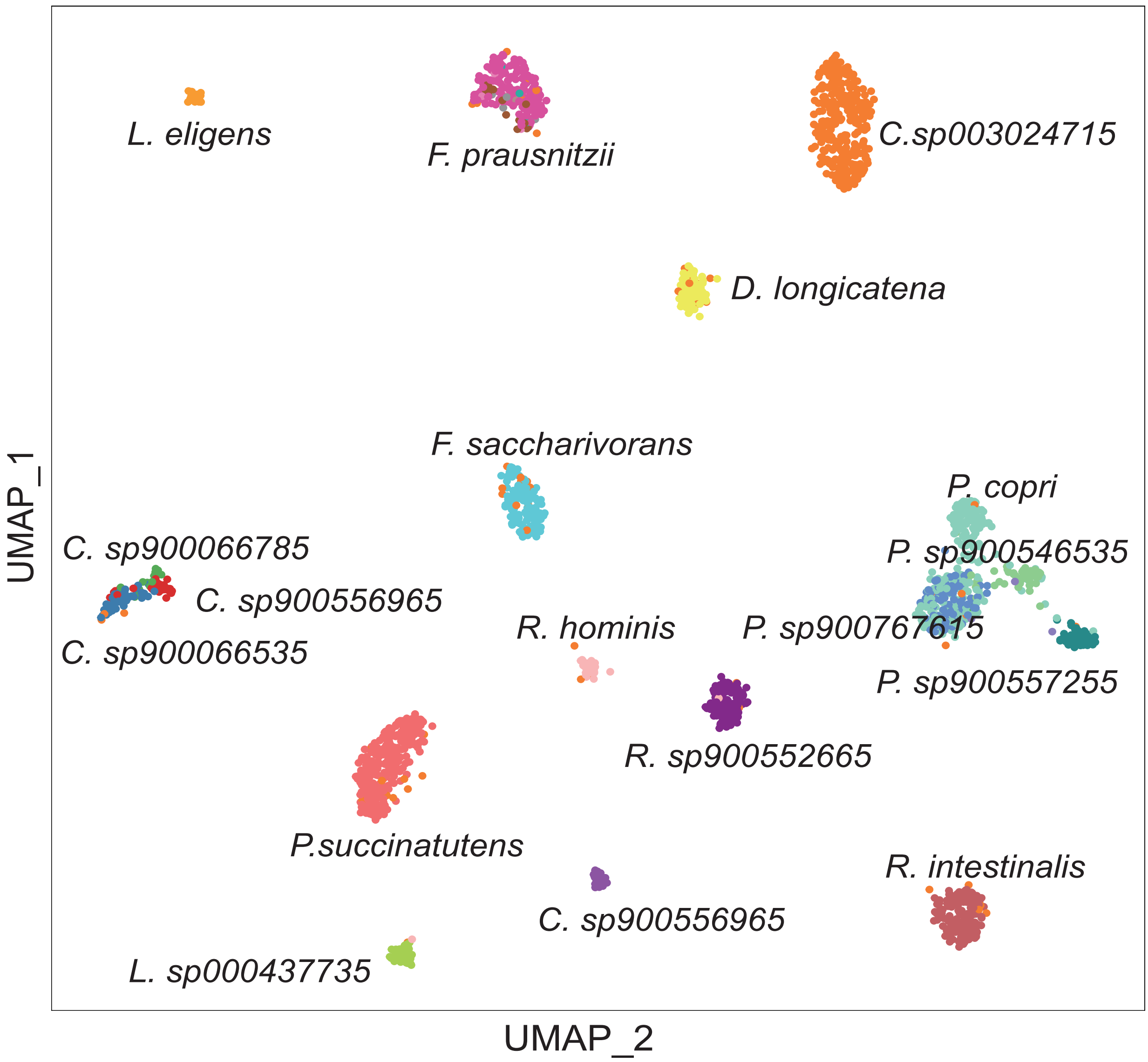

Figure S6. Host-phage transcriptional relationship of human gut microbiome at species level. The bacteria of major genera (≥ 3% sample proportion) were extracted and clustered with species scale taxonomic information from MIC-anno for UMAP projection.

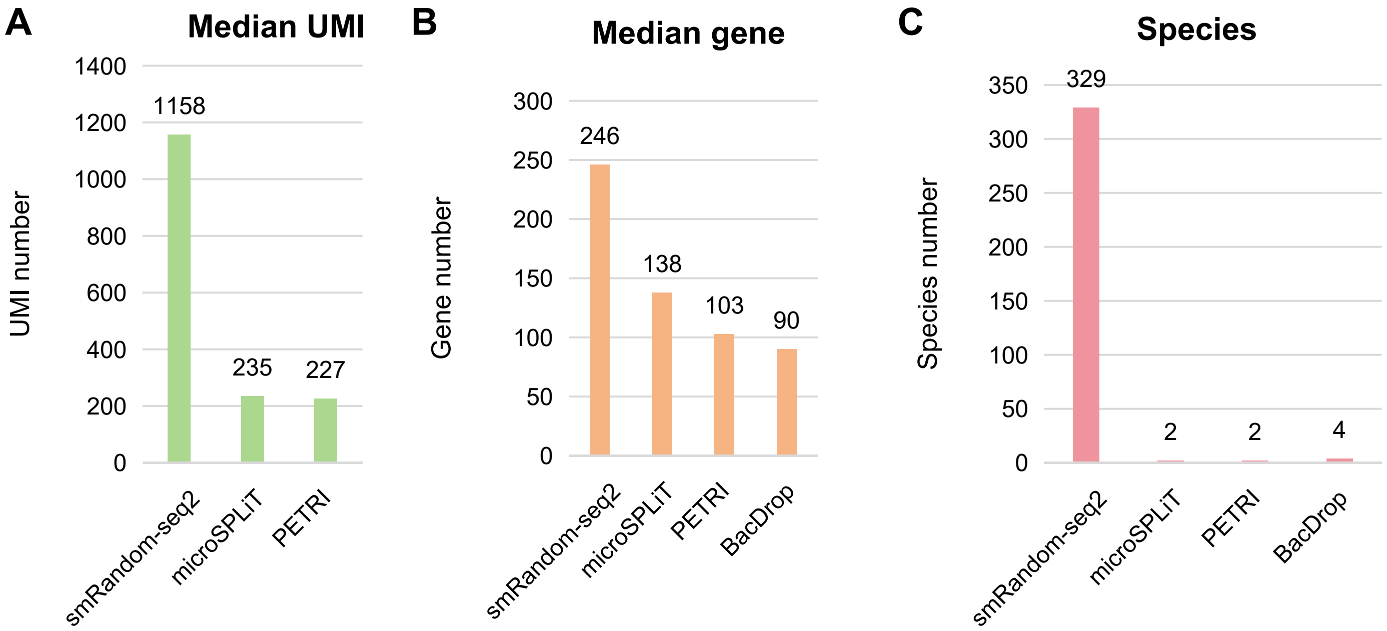

Figure S7. **Comparison of smRandom-seq2 to other available techniques.** Comparison of the UMI reads number (A), gene number (B), and species number (C) by smRandom-seq2 to other available techniques.

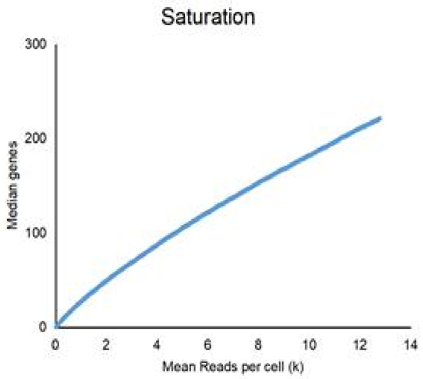

Figure S8. **Saturation analysis of smRandom-seq2**.

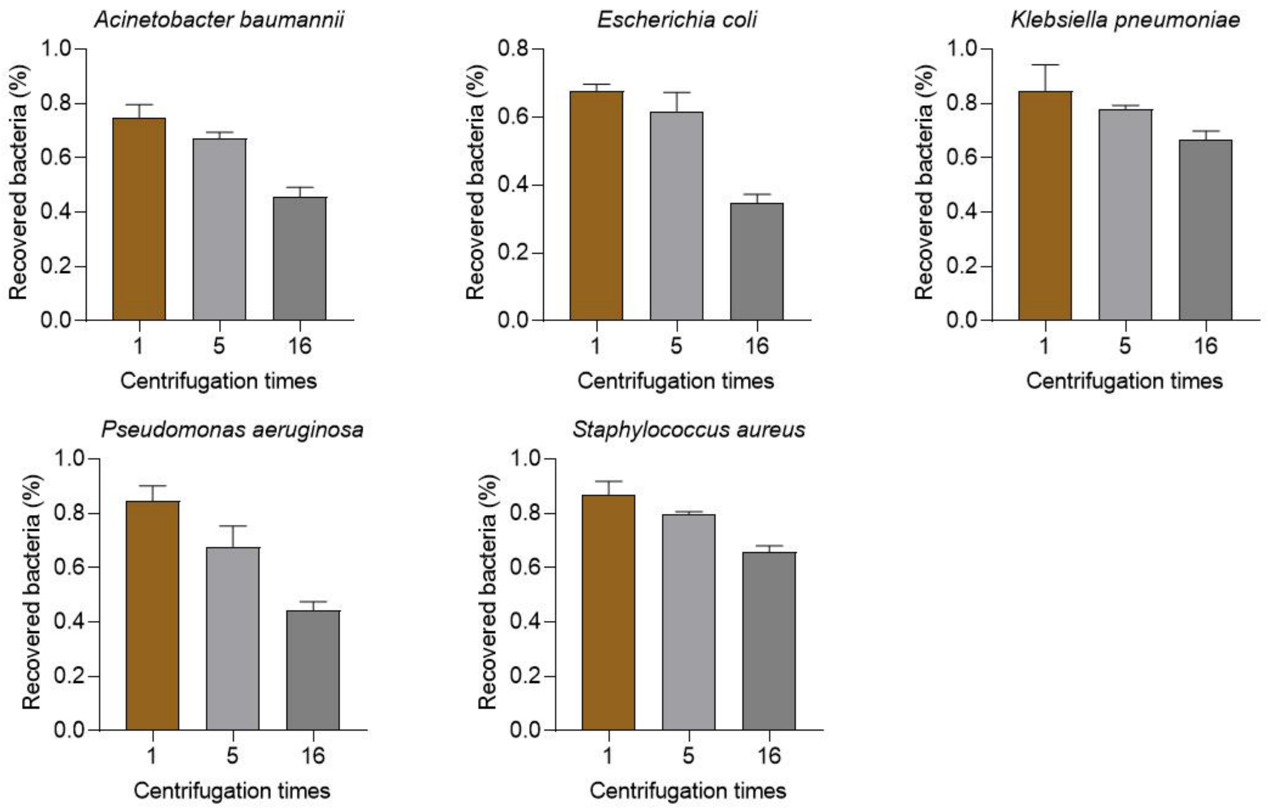

Figure S9. **The recovered rate of the bacteria with different centrifugation times.**
